# Lutein Modulates Oxidative Stress, Inflammatory and Apoptotic Biomarkers Related to Di-(2-Ethylhexyl) Phthalate (DEHP) Hepato-Nephrotoxicity in Male Rats: Role of Nuclear Factor Kappa B

**DOI:** 10.3390/toxics11090742

**Published:** 2023-08-30

**Authors:** Dina R. S. Gad El-Karim, Mohamed A. Lebda, Badriyah S. Alotaibi, Attalla F. El-kott, Heba I. Ghamry, Mustafa Shukry

**Affiliations:** 1 Department of Pathology and Clinical Pathology, Faculty of Veterinary Medicine, Alexandria University, Alexandria 22758, Egypt; 2 Department of Biochemistry, Faculty of Veterinary Medicine, Alexandria University, Alexandria 22758, Egypt; 3 Department of Pharmaceutical Sciences, College of Pharmacy, Princess Nourah bint Abdulrahman University, P.O. Box 84428, Riyadh 11671, Saudi Arabia; 4 Department of Biology, College of Science, King Khalid University, Abha 61421, Saudi Arabia; 5 Department of Zoology, Faculty of Science, Damanhour University, Damanhour 22511, Egypt; 6 Nutrition and Food Sciences, Department of Home Economics, Faculty of Home Economics, King Khalid University, P.O. Box 960, Abha 61421, Saudi Arabia; 7 Physiology Department, Faculty of Veterinary Medicine, Kafrelsheikh University, Kafrelsheikh 33516, Egypt

**Keywords:** phthalates, lutein, hepato-nephrotoxicity, rats, Nf-κB

## Abstract

Phthalates are widely distributed in our environment due to their usage in many industries, especially in plastic production, which has become an essential part of daily life. This investigation aimed to assess the potential remedial influence of lutein, a naturally occurring carotenoid, on phthalate-triggered damage to the liver and kidneys. When di-(2-ethylhexyl) phthalate (DEHP) was administered to male albino rats over sixty straight days at a dosage of 200 mg/kg body weight, it resulted in a significant increase in the serum activity of liver enzymes (AST, ALT, and GGT), alpha-fetoprotein, creatinine, and cystatin-C, as well as disruptions in the serum protein profile. In addition, intoxication with DEHP affected hepato-renal tissues’ redox balance. It increased the content of some proinflammatory cytokines, nuclear factor kappa B (Nf-κB), and apoptotic marker (caspase-3); likewise, DEHP-induced toxicity and decreased the level of anti-apoptotic protein (Bcl-2) in these tissues. Lutein administration at a dose level of 40 mg/kg b.w efficiently facilitated the changes in serum biochemical constituents, hepato-renal oxidative disturbance, and inflammatory, apoptotic, and histopathological alterations induced by DEHP intoxication. In conclusion, it can be presumed that lutein is protective as a natural carotenoid against DEHP toxicity.

## 1. Introduction

Phthalates (phthalic acid derivatives) are synthetic chemicals that are widely produced for usage in many industries. They are the main part of the plastic industry as plasticizers due to their ability to enhance plastic materials’ durability, transparency, and flexibility. So, the phthalates group is one of the most abundant environmental contaminants [1]. In addition, phthalates could be used as solvents in various products, including paints and insect sprays, and as color and scent stabilizers in cosmetics [2]. Massive usage of phthalates leads to exposure of humans and animals to their toxicity via ingestion, dermal absorption, and inhalation [3]. Still, ingestion is the main route of exposure [4]. Di-(2-ethylhexyl) phthalate (DEHP) is the main phthalate derivative that is used in the production of polyvinyl chloride (PVC). Unfortunately, DEHP leaks easily from PVC due to its weak bond with plastic material, contributing to its health hazard effect [5]. DEHP and its metabolites are linked with a wide range of adverse effects on the kidney, liver, heart, lung, and reproductive tract [6]. The presence of phthalate derivative metabolites was proved in all tested human urine samples by Heudorf et al. [7]. Different mechanisms are implicated in the induction of phthalates toxicity, but oxidative stress is the most acceptable [8]. The metabolism of DEHP occurs in various tissues containing hydrolase, unspecific lipase, and esterase enzymes, producing mono-2-ethylhexyl) phthalate and 2-ethylhexanol [9]. These metabolized products disrupt mitochondrial function and release cytochrome c, leading to the generation of ROS and decreased antioxidant capacity [10]. DEHP induces liver injury by promoting the production of ROS and activating LPO, resulting in lipid peroxidation and damage to the cell structure and function of the liver [11]. Zhang et al. [12] found that excessive ROS production due to DEHP treatment affects MDA and SOD levels, leading to cell lipid accumulation and potential cell death. Excessive ROS reacts with fatty acids to form LPO products like MDA and HNE, causing cell membrane fluidity and permeability changes, leading to cell structure and function damage [13]. On the other hand, lutein is a natural carotenoid present abundantly in green and colored vegetables and fruits [14]. Lutein has many pharmacological and biological properties, including antioxidant [15], anti-inflammatory [16], hepato-protective [17], nephroprotective [18], cardio-protective [19], and anti-cancer [20] effects. In this consistency, this study aimed to evaluate the toxic impact of di-(2-ethylhexyl) phthalate on the liver and kidney and the presumptive ameliorative role of lutein as a natural carotenoid against this toxicity.

## 2. Material and Methods

### 2.1. Chemicals

Di (2-ethylhexyl) phthalate (DEHP, ±99% purity) was purchased from Sigma–Aldrich (St Louis, MO, USA). Lutein was obtained as capsules containing 20 mg of lutein ((Des Moines, IA, USA). Biochemical diagnostic kits were from Spinreact Kits, Spain; Cusabio^®^, Wuhan, China; Crystal Chem, Elk Grove Village, IL, USA; and Biodiagnostic Co, Giza, Egypt.

### 2.2. Experimental Animals and Design

Twenty-eight male albino rats (180–200 g) were purchased from Pharos University Animal House, Alexandria, Egypt, to accomplish this study. They were kept in separate metal cages at ambient humidity and temperature; a 12/12 h light/darkness cycle was applied. Basal experimental diet and water were offered ad libitum; they were kept without any treatment for ten days to acclimatize and to ensure they were free from any apparent health problems. After acclimatization, they were divided into four equal groups (7 rats/each): the control group received corn oil 1 mL/kg orally; the lutein group received lutein at a dose level of 40 mg/kg b.w; DEHP group intubated with DEHP at a dose level of 200 mg/kg b.w; DEHP/lutein group received both DEHP (200 mg/kg b.w) [21,22,23,24] and lutein (40 mg/kg b.w) [25].

All the treatments were administrated orally after dissolving in corn oil using gastric gavage. The treatment protocols were administrated daily for sixty consecutive days. The animals were sacrificed through cervical dislocation after deep anesthesia with ketamine/xylazine (7.5–10 mg/kg, 1 mg/kg i.p) 24 h. following the last administration of the treatment protocols.

### 2.3. Sampling and Biochemical Analysis

#### 2.3.1. Serum Biochemical Analysis

Blood samples were obtained from the heart directly using a syringe after dissection of the sacrificed animals. The blood was drained in plain tubes, left to coagulate for 30 min at room temperature, and then centrifuged at 3000 rpm for 10 min to separate serum. Serum aliquots were kept at −20 °C for further detection of serum activities of hepatic transaminase enzymes (AST and ALT), gamma-glutamyl transferase enzyme (GGT), in addition to the serum concentration of creatinine, total protein, and albumin (Spinreact kits, Barcelona, Spain). Also, serum levels of alpha-fetoprotein (AFP) (Cusabio^®^, Wuhan, China) and cystatin-C (Crystal Chem, 955 Busse Rd, Elk Grove Village, IL 60007, USA) were detected.

#### 2.3.2. Preparation of Tissue Homogenate

The left kidney of each animal, in addition to one lobe of the liver, was obtained, washed several times with phosphate buffer saline (PBS), and jabbed with PBS containing heparin to remove any blood clots. Samples were then dissected into small pieces using a scalpel, and PBS was added to obtain 10% tissue homogenate using a tissue homogenizer (Glas-Col^®^, Beijing, China). The obtained homogenates were centrifuged at 3000 rpm for 20 min and filtrated; the clear supernatant was kept at −80 °C for further evaluation of oxidant/antioxidant, inflammatory, and apoptotic biomarkers. The protein content of homogenate was detected using Bradford’s reagent Sigma-Aldrich (St. Louis, MO, USA).

#### 2.3.3. Evaluation of Oxidant/Antioxidant Biomarkers

The levels of malondialdehyde (MDA), reduced glutathione (GSH), and the activity of the catalase enzyme (CAT) in the liver and kidneys were measured utilizing kits commercially sourced from Biodiagnostic, Egypt.

#### 2.3.4. Evaluation of Inflammatory and Apoptotic Biomarkers

Hepatic and renal levels of interleukin-1beta (IL-1β), tumor necrosis factor-alpha (TNF-α) (Abcam, Cambridge, MA, USA), Bcl-2, caspase-3 and nuclear factor kappa B (Cusabio^®^, Wuhan, China) were determined using previously listed highly specific ELISA-based kits.

#### 2.3.5. Histopathological Examination

The right kidney of each animal and small pieces of liver were removed and washed several times with normal saline and kept in 10% formalin solution to perform a 5 µm thickness tissue section for staining with H&E, according to Bancroft and Stevens [26]

#### 2.3.6. Histopathological Semi-Quantitative Scoring System

Five random fields (×100) were randomly selected from each rat in each group. The present pathological lesions were detected and scored as follows according to the severity of the presented lesions, which were achieved relying on the percentage of the affected area/entire section: - = absence of lesion, + (mild) = 5-25%, ++ (moderate) = 26-50%, and +++ (severe) = ≥50%.

#### 2.3.7. Statistical Analysis

A one-way analysis of variance (ANOVA) test, executed through the Statistical Analysis System (SAS) software, was employed to investigate the influence of distinct treatment methods on the parameters assessed.

## 3. Results

### 3.1. Serum Findings of Liver and Kidney Functions

As present in Table 1, serum activities of hepatic enzymes AST (+80%), ALT (+120%), and GGT (+311%), in addition to the serum level of AFP (+120%), globulin (+37%) and renal biomarker; cystatin-C (+150%) were significantly incriminated in DEHP-intoxicated rats, while serum level of albumin (−26%) was decreased in the same group when compared to control rats. These changes were facilitated significantly in rats pretreated with lutein, improving the liver and renal biomarkers.

### 3.2. Hepato-Renal Redox State

Intoxication with DEHP significantly increased the content of lipid peroxide (MDA) in both hepatic (+87%) and renal (+138%) tissues, depleted the concentration of GSH (−50%, −36%, respectively), and the activity of CAT enzyme within these tissues in comparison with a control group. However, co-treatment with lutein restored the oxidative balance of these tissues partially, as the level of MDA was decreased (~23% in both tissues). The activity of CAT was increased (~58% in the liver and ~38% in the kidneys), alongside significant elevation in the GSH content (~55% in the liver and ~29% in kidneys), as represented in Table 2.

### 3.3. Proinflammatory Cytokines and Apoptotic Biomarkers

**Figure 1:**
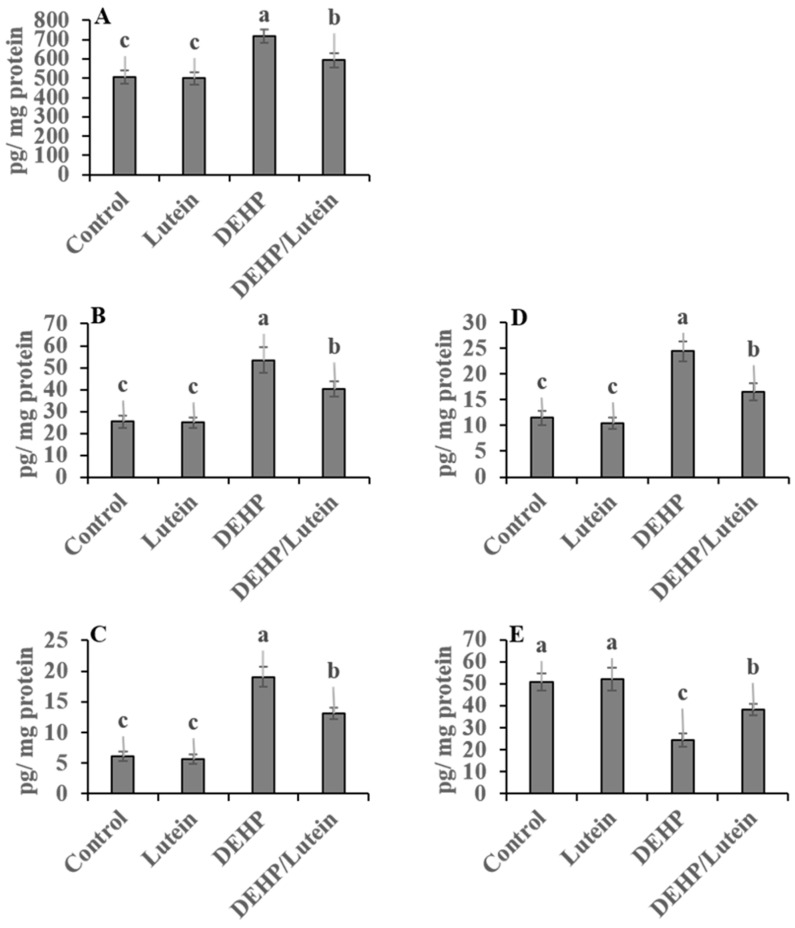
Hepatic proinflammatory and apoptotic biomarkers in DEHP and lutein-treated groups. (**A**); nuclear factor-κβ (NF-κβ), (**B**); tumor necrosis factor-α (TNF-α), (**C**); interleukin-1β (IL-1β), (**D**); caspase-3, (**E**); β cell lymphoma-2 (Bcl2). Results are displayed as Mean ± Standard Error. ^a,b,c^ Noteworthy differences (*p* < 0.05) are observed among the columns with distinct litters.

**Figure 2:**
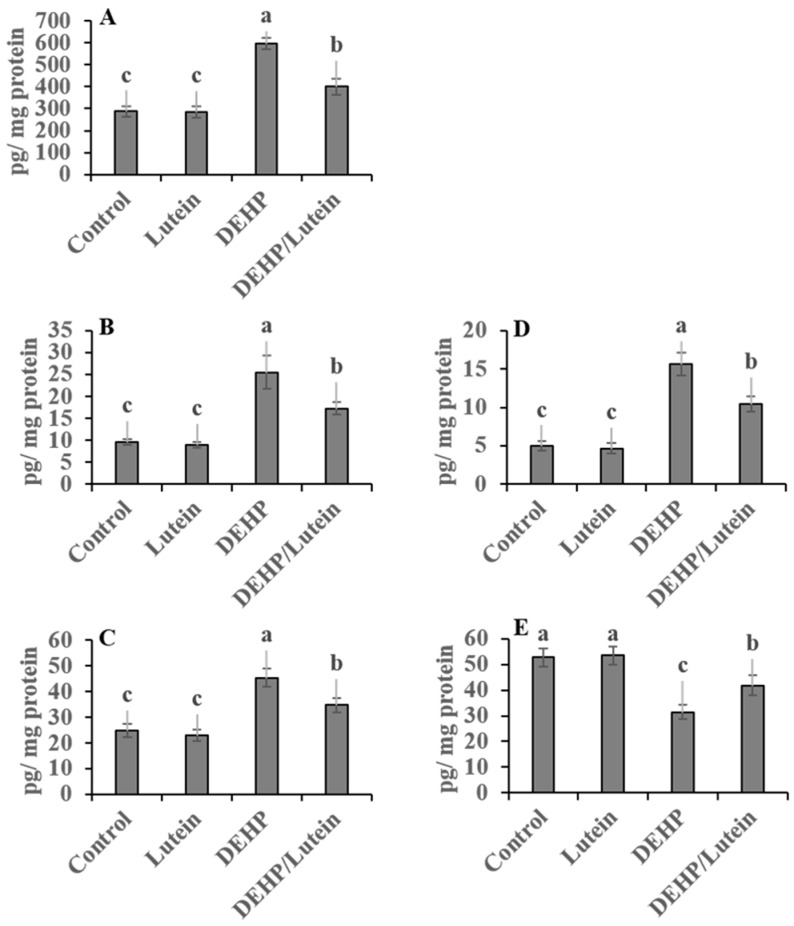
Renal proinflammatory and apoptotic biomarkers in DEHP and lutein-treated groups. (**A**); nuclear factor-κβ (NF-κβ), (**B**); tumor necrosis factor-α (TNF-α), (**C**); interleukin-1β (IL-1β), (**D**); caspase-3, (**E**); β cell lymphoma-2 (Bcl2). Results are displayed as Mean ± Standard Error. ^a,b,c^ Noteworthy differences (*p* < 0.05) are observed among the columns with distinct litters.

Oral administration of DEHP statistically elevated hepatic and renal proinflammatory levels; TNF-α (~112% and 177%), IL-1β (~280% and 87%), NF-κβ (~41% and 107%), with augmented the proapoptotic marker; caspases-3 (~118% and 200%), while reducing the anti-apoptotic index level; Bcl-2 (~52% and 41%) as compared to control group indicating inflammatory and apoptotic conditions observed in both tissues with the kidney is predominant. Lutein administration simultaneously with DEHP significantly has anti-inflammatory activity, decreasing the proinflammatory cytokines and proapoptotic biomarkers in hepato-renal tissues. It also enhanced the anti-apoptotic index in both tissues, as recorded in Figure 1 and Figure 2.

### 3.4. Histopathological Changes

#### 3.4.1. Liver

**Figure 3:**
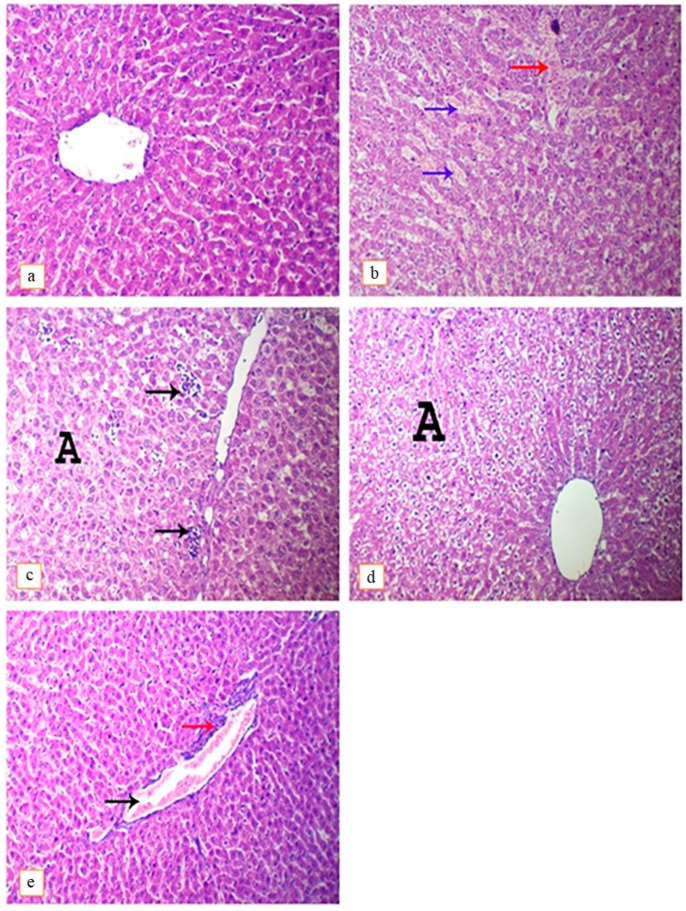
Photomicrograph of rat’s liver intoxicated with DEHP and treated with lutein, H&E. (×400). (**a**); control group, showing normal histoarchitecture, (**b**); DEHP-intoxicated group, showing congestion of hepatic sinusoids (blue arrows), hemorrhage (red arrow), (**c**); DEHP-intoxicated group, showing multi-focal hepatic necrosis (arrows) and hydropic degeneration of hepatocytes (A), (**d**); DEHP and lutein co-treated group, showing hydropic degeneration of hepatocytes (A), (**e**); DEHP and lutein co-treated group, showing congestion of blood vessel (black arrow) and perivascular infiltration of mononuclear inflammatory cells (red arrow).

Upon histopathological examination, the liver of the control group appeared with normal histoarchitectures (Figure 3a). In contrast, hepatic tissues of the DEHP-intoxicated group showed congestion of hepatic sinusoids with hemorrhage (Figure 3b), in addition to widespread hepatocytes hydropic degeneration and the presence of necrotic foci (Figure 3c). Lutein co-treated with DEHP showed only low-grade hydropic degeneration of hepatocytes (Figure 3d) and congestion of blood vessels, which was accompanied by perivascular inflammatory cell infiltration (Figure 3e).

#### 3.4.2. Kidney

**Figure 4:**
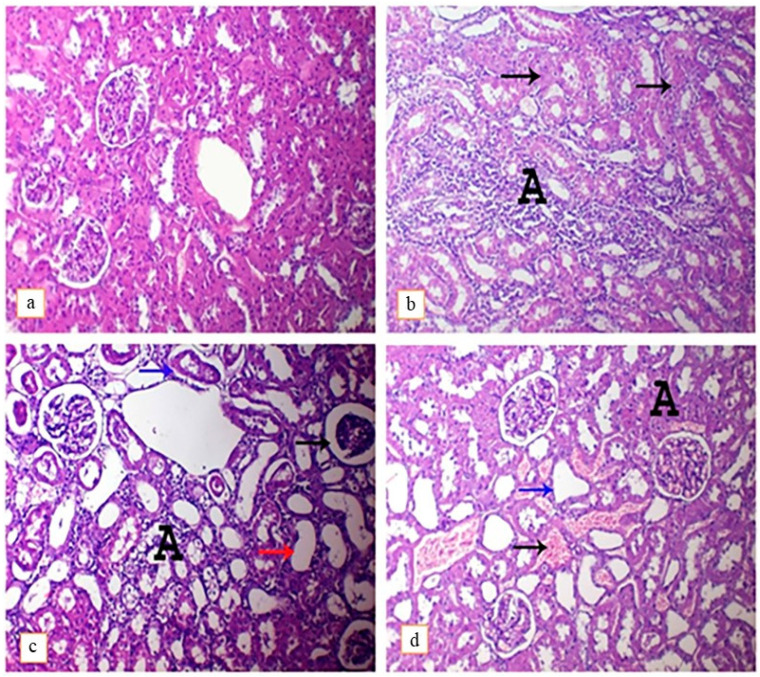
Photomicrograph of rat’s kidneys intoxicated with DEHP and treated with lutein, H&E. (×400). (**a**); control group, showing normal histoarchitecture, (**b**); DEHP-intoxicated group, showing widespread interstitial mononuclear inflammatory cells infiltration (A) and tubular necrosis (arrows), (**c**); DEHP-intoxicated group, showing atrophied glomerulus (arrows), hydropic degeneration of tubular epithelium with interstitial inflammatory cells infiltration (A), cystic dilatation of renal tubules (red arrow) and presence of detached renal epithelium within the lumen of renal tubules (blue arrow), (**d**); DEHP and lutein co-treated group, showing congestion of interstitial blood vessels (black arrow) with focal necrosis of renal tubules (A) and cystic dilation of renal tubules.

Histopathological examination of renal tissues of the control group revealed the presence of normal glomeruli and renal tubules without any detected lesions (Figure 4b). On the contrary, DEHP-intoxicated rats showed the presence of interstitial inflammatory cells infiltration and tubular necrosis (Figure 4b), with vacuolar degeneration of tubular epithelium, presence of atrophied glomeruli, detached tubular epithelium and cystic dilatation of renal tubules (Figure 4c). On the other hand, administration of lutein with DEHP only causes congestion of interstitial blood vessels with mild focal tubular necrosis and cystic dilation (Figure 4d). The ameliorative effect of lutein administration against DEHP-induced hepato-nephrotoxicity-related lesions has been reflected in scores of these detected lesions, as shown in Table 3.

## 4. Discussion

DEHP has attracted attention during the last few years as one of the most dangerous environmental toxicants due to its wide usage in the production of polyvinyl chloride (PVC)-based products such as food containers, water pipes, and medical devices which can be incriminated in human and animal toxicity [27]. Most environmental toxicant exerts a deleterious effect on the body’s organs through the generation of ROS [28], which could disturb cell functions, causing cell death and even carcinogenesis [29]. Concerning DEHP-induced hepatotoxicity, several studies have indicated that such toxicity is strongly related to redox balance disturbance and depletion of tissue antioxidants [30]. In this context, the increase in serum activity of hepatocytic transaminase enzymes (AST and ALT) and cholestatic enzyme (GGT) confirmed the toxic effect of DEHP on the liver, as reported before by Erkekoglu et al. [31]; these enzymes are released from hepatocytes and biliary epithelium lining respectively upon their destruction which led to their increase in serum [32]. DEHP’s carcinogenic effect has been studied extensively [33], and the liver is one of the most predilection sites for DEHP carcinogenicity [34]. AFP is one of the major tumor-associated proteins [35] produced by the liver, and its production is increased in response to carcinogenesis, especially hepatocellular carcinoma [36]. So, the detected increase in serum level of AFP upon exposure to DEHP may indicate its carcinogenic effect on the liver. DEHP nephrotoxicity has been reported and confirmed in animals and cellular models [37]. Similarly, DEHP-related nephrotoxicity may be owed to DEHP-induced ROS production [38]. So, the detected elevation in serum level of creatinine and cystatin-C (kidney functional biomarkers) would confirm DEHP’s nephrotoxic effect as reported before [23]. Albumin is one of the negative acute phase proteins (which decrease in response to inflammation), and globulins are positive acute phase proteins (which increase in response to inflammation); acute phase proteins are increased or decreased due to the release of inflammatory cytokines [39], and this may explain the decrease in serum albumin level and increased serum globulins level in DEHP-intoxicated animals, in response to hepato-renal inflammatory state which will be discussed later. Also, defective hepatic albumin production or renal albumin loss due to DEHP-induced hepatorenal toxicity could be another explanation for decreased albumin levels [32]. In our study, the previously discussed DEHP-induced redox homeostasis disturbance in renal and hepatic tissues was reflected in the form of an elevation of MDA content in these tissues and depletion of their content of enzymatic and non-enzymatic defensive antioxidants (CAT, SOD, and GSH). Production of ROS may induce cell death due to the activation of different pathways and factors, including inflammatory and apoptotic factors [40]. The latter may explain the significant increase in the renal and hepatic levels of proinflammatory cytokines (IL-1β and TNF-α), as the increase in ROS production would enhance the inflammatory process and stimulate the release of proinflammatory cytokines. These cytokine releases could induce further ROS production [41]. Nf-κB plays a crucial role in the induction of inflammation, as it can increase the transcription of proinflammatory cytokines and chemokines [42]. Another theory for DEHP-induced toxicity includes increased production of Nf-κB in affected tissues [23], as reported in our study. Also, Huang et al. [43] reported upregulation of proinflammatory cytokines (NF-κβ, IL-6, IL-1β, and TNF-α) upon exposure of quail to DEHP. Fortunately, as a part of the anti-inflammatory activity of carotenoids, lutein’s ability to reduce Nf-κB was proved before [44], so the level of inflammatory Nf-κB was decreased in hepatic and renal tissues of DEHP-intoxicated rats treated with lutein. Apoptosis is strongly implicated in DEHP-induced toxicity [45], which is mediated by several apoptotic proteins such as caspase-3, and this may explain the increase in hepatic and renal tissue content of caspase-3 (apoptotic protease). Also, the elevation in the caspase-3 level may be attributed to the decrease in Bcl-2 (anti-apoptotic protein), which is responsible for the regulation of the caspase-3 level [46]. Previously discussed biochemical changes related to DEHP hepato-renal toxicity were associated with variable lesions in both hepatic and renal tissues upon histopathological evaluation. Lutein antioxidant, anti-inflammatory, and anti-apoptotic activities were detected and proven previously [47]. So, based on these activities, its role in the amelioration of hepato-renal injuries and toxicity was proved in several studies [18]. Similarly, in our study, lutein successfully mitigated DEHP-induced hepato-renal toxicity and its related biochemical, inflammatory, and apoptotic biomarkers alterations; the lutein ameliorative effect was confirmed histo-pathologically through lowering the score of the induced lesions in hepatic and renal tissues. The structural base of the antioxidative effect of lutein is believed to contribute to the delocalization of unpaired electrons by its conjugated double-bonded structure. This allows lutein to effectively scavenge free radicals such as superoxide, hydroxyl, and peroxyl radicals. By scavenging these free radicals, lutein helps prevent the generation of oxidative stress, which can lead to cellular damage. This is why lutein is often considered a potent antioxidant with potential health benefits [48]. Finally, it could be concluded that lutein, as one of the natural ingredients of our daily food, can abrogate DEHP-induced hepato-nephrotoxicity due to its potent antioxidant, anti-inflammatory, and anti-apoptotic effects.

## 5. Conclusions

This study demonstrates that exposure to DEHP resulted in a significant increase in liver enzyme activity and alpha-fetoprotein, creatinine, cystatin-C, and disruptions in serum protein profile. Furthermore, DEHP intoxication disrupted the redox balance in hepato-renal tissues, leading to an increase in proinflammatory cytokines, nuclear factor kappa B (Nf-κB), and apoptotic marker (caspase-3) while decreasing the level of anti-apoptotic protein (Bcl-2) in these tissues. However, administering lutein at a dose of 40 mg/kg b.w efficiently facilitated the changes in serum biochemical constituents, hepato-renal oxidative disturbance, inflammatory and apoptotic markers, and histopathological alterations induced by DEHP intoxication. As a natural carotenoid, lutein is likely to protect against DEHP toxicity.

## Figures and Tables

**Table 1:** Serum biochemical findings of liver and kidney functions in DEHP and lutein-treated groups.

	Control	Lutein	DEHP	DEHP/Lutein
AST (U/L)	115.00 ^c^ ± 7.12	114.71 ^c^ ± 7.48	209.29 ^a^ ± 7.85	166.86 ^b^ ± 5.45
ALT (U/L)	24.14 ^c^ ± 2.26	23.57 ^c^ ± 1.86	53.29 ^a^ ± 3.39	41.00 ^b^ ± 1.98
GGT (U/L)	18.14 ^c^ ± 2.18	17.64 ^c^ ± 1.96	74.14 ^a^ ± 4.67	47.57 ^b^ ± 4.37
AFP (ng/mL)	5.11 ^c^ ± 0.64	4.90 ^c^ ± 0.55	11.50 ^a^ ± 1.15	7.54 ^b^ ± 0.47
Cystatin-C (mg/L)	2.58 ^c^ ± 0.33	2.47 ^c^ ± 0.29	5.82 ^a^ ± 0.40	3.87 ^b^ ± 0.35
Total protein (g/dL)	6.36 ± 0.12	6.37 ± 0.13	6.16 ± 0.15	6.41 ± 0.16
Albumin (g/dL)	3.83 ^a^ ± 0.13	3.87 ^a^ ± 0.13	2.80 ^b^ ± 0.13	3.51 ^a^ ± 0.14
Globulins (g/dL)	2.51 ^c^ ± 0.09	2.50 ^c^ ± 0.05	3.44 ^a^ ± 0.11	2.94 ^b^ ± 0.06

Results are displayed as Mean ± Standard Error. ^a,b,c^ Noteworthy differences (*p* < 0.05) are observed among the means within the same rows of distinct litters. AST; aspartate aminotransferase, ALT; alanine aminotransferase, GGT; gamma-glutamyl transferase, AFP; alfa fetoprotein, DEHP; di-(2-ethylhexyl) phthalate.

**Table 2:** Hepato-renal oxidant/antioxidant biomarkers in DEHP and lutein-treated groups.

	Control	Lutein	DEHP	DEHP/Lutein
Hepatic oxidant/antioxidative indices				
MDA (nmol/mg protein)	117.00 ^c^ ± 8.31	119.00 ^c^ ± 9.06	219.86 ^a^ ± 10.90	171.57 ^b^ ± 12.25
GSH (nmol/mg protein)	18.86 ^a^ ± 1.79	18.21 ^a^ ± 0.98	9.43 ^c^ ± 0.62	14.24 ^b^ ± 1.04
CAT (U/mg protein)	26.71 ^a^ ± 1.82	27.86 ^a^ ± 1.75	12.07 ^c^ ± 1.21	19.00 ^b^ ± 1.50
Renal oxidant/antioxidative indices				
MDA (nmol/mg protein)	50.57 ^c^ ± 3.64	52.14 ^c^ ± 3.62	119.14 ^a^ ± 7.23	91.86 ^b^ ± 4.61
GSH (nmol/mg protein)	44.43 ^a^ ± 2.40	45.00 ^a^ ± 1.94	28.14 ^c^ ± 2.59	36.29 ^b^ ± 1.96
CAT (U/mg protein)	4.20 ^a^ ± 0.45	4.23 ^a^ ± 0.50	2.61 ^c^ ± 0.25	3.69 ^b^ ± 0.31

Results are displayed as Mean ± Standard Error. ^a,b,c^ Noteworthy differences (*p* < 0.05) are observed among the means within the same rows of distinct litters. DMA; malondialdehyde, GSH; reduced glutathione, CAT; catalase.

**Table 3:** Semi-quantitative scoring results of the detected hepato-renal lesions.

Incidence ^1^ and Severity ^2^ of Histopathological Lesions								
	DEHP Intoxicated Rats				DEHP and Lutein-Treated Rats			
	Absent (-)	Mild (+)	Moderate (++)	Severe (+++)	Absent (−)	Mild (+)	Moderate (++)	Severe (+++)
	Liver							
1-Hydropic degeneration	0	2	0	5	3	1	2	1
2-Congested sinusoids	2	2	2	1	5	2	0	0
3-Congestion of blood vessels	1	2	2	2	2	2	1	2
4-Perivascular infiltration of inflammatory cells	3	1	2	1	3	2	1	1
4-Necrotic foci	1	3	1	2	3	4	0	0
5-Hemorrhage	2	1	2	2	6	0	1	0
	Kidney							
1-Atrophied glomeruli	3	1	2	1	5	2	0	0
2-Congested blood vessels	2	2	1	2	4	1	1	1
2-Necrotic tubules	0	2	2	3	2	2	1	2
3-Interstitial nephritis	2	1	1	3	4	2	1	0
4-Hydropic degeneration of tubular epithelium	3	3	1	0	4	2	0	1
5-Cystic dilatation	1	3	1	2	3	1	1	2
6-Detached tubular epithelium	2	1	2	2	5	1	1	0

^1^ Number of rats with lesions per total examined (7 rats). ^2^ Severity of lesions was graded by estimating the percentage area affected in the entire section.

## Data Availability

Upon request.
